# Editorial: Emerging Proteins and Polypeptides Expressed by “Non-Coding RNAs”

**DOI:** 10.3389/fcell.2022.862870

**Published:** 2022-02-21

**Authors:** Wanting Liu, Qing-Yu He, Marie A. Brunet

**Affiliations:** ^1^ MOE Key Laboratory of Tumor Molecular Biology and Key Laboratory of Functional Protein Research of Guangdong Higher Education Institutes, Institute of Life and Health Engineering, Jinan University, Guangzhou, China; ^2^ Department of Pediatrics, Medical Genetics Service, Université de Sherbrooke, Sherbrooke, QC, Canada; ^3^ Centre de Recherche du Centre Hospitalier Universitaire de Sherbrooke, Sherbrooke, QC, Canada

**Keywords:** non-coding RNA (ncRNA), alternative ORFs, small ORFs, MicroProtein, ribosome profiling (RIBO-Seq), mass spectrometry

By definition, non-coding RNAs (ncRNAs) are RNA molecules that do not encode proteins. Yet, emerging evidences, drawn from deep ribosome sequencing and mass spectrometry, show that a subset of ncRNAs including long non-coding RNAs (lncRNAs) and cirRNAs are able to encode functional proteins/polypeptides (Makarewich and Olson, 2017; Orr et al., 2020; Peeters and Menschaert, 2020). Although the function of these novel proteins remains sometimes elusive, some have been demonstrated to play vital functions in human health. The identification and functional characterization of these novel proteins is a new emerging field of biological sciences. Recent studies have shown that these novel proteins are involved in diverse biological functions such as mitochondrial function (Chen et al., 2018; Stein et al., 2018), lipid metabolism (Chibucos et al., 2014; Chen et al., 2018; Polycarpou-Schwarz et al., 2018; Singh et al., 2018; Zhang et al., 2019; Zhang et al., 2020), tumor energy metabolism (Chibucos et al., 2014; Kim et al., 2021), cell development (Kulczynska and Siatecka, 2016; Fazi and Fatica, 2019; Attaway et al., 2021; Kersy et al., 2021; Kim et al., 2021), and DNA repair (Sharma and Misteli, 2013; Slavoff et al., 2014; Zhou et al., 2015; Dianatpour and Ghafouri-Fard, 2017; Thapar, 2018; Attaway et al., 2021; Papaspyropoulos et al., 2021). This research topic in Frontiers in Cell and Developmental Biology focused on recent progress in this emerging field, aiming to better understand “ncRNAs,” and served as a forum to discuss gene annotation and the discovery of novel physiological and pathological molecules.

## Non-Coding RNAs: An Overlooked Source of Functional Proteins

Non-coding RNAs have recently been demonstrated to contain small-open reading frames (sORFs) encoding small proteins. Only a few of these newly discovered proteins have been functionally characterized so far, but they are key players in a variety of cellular processes. In this topic, authors have reviewed or provided new evidence for the overlooked coding potential of some lncRNAs. The collection of article illustrates the diversity of functions of these novel proteins, from glioblastoma biomarkers to neuropeptides and regeneration.

In an extensive review, Cardon et al. discuss lncRNAs-encoded proteins as novel biomarkers for glioblastoma (GBM). They review evidence linking these to the patient’s survival and bad prognosis. The authors also highlighted the potential functions of these novel proteins in GBM biology by showing their interaction with known proteins in the signaling pathways of cellular mobility and transfer RNA regulation.

Novel proteins originating from lncRNAs have been found in many biological samples, representing a variety of tissues and cell types. To better understand the role of ncRNAs-encoded microproteins in different tissues, Pan et al. profiled the proteomes of five mouse tissues by mass spectrometry with bottom-up, top-down, and *de novo* sequencing strategies. Using the OpenProt database (Brunet et al., 2019; Brunet et al., 2021), they identified 1,074 microproteins, 540 were known and 534 were novel, including 270 from ncRNAs. They performed gene ontology analyses on the 540 already annotated microproteins to highlight tissue-specific functions. For example, the brain contains the largest number of neuropeptides, and the spleen contains the most immune-associated microproteins. Their results expand the mouse proteome and provide insights into the molecular biology of mouse tissues.

Working with mouse embryonic stem cells, Senís et al. discovered a conserved microprotein, named pTUNAR, encoded in the *TUNAR* lncRNA. The authors showed that the 48 amino-acid long pTUNAR is expressed in the nervous system using ribosome profiling and a custom antibody. They identified pTUNAR at the membrane of the endoplasmic reticulum, in interaction with SERCA2. Their results validate the previous work of Li et al. (2021) where pTUNAR was independently identified (and named BNLN) and found in interaction with SERCA3. Although further work is needed to understand how pTUNAR regulates calcium dynamics, this work confirmed previous findings and suggest pTUNAR as an important player in neural differentiation and neurite formation.

Another type of non-coding RNA are telomerase RNA. Along with the telomerase reverse transcriptase and regulatory proteins, it makes up the telomerase complex. However, telomerase RNA is expressed in most somatic cells, whereas the telomerase reverse transcriptase is absent. This observation prompted Rubtsova et al. (2018) to investigate the coding potential of human telomerase RNA and discovered the human telomerase RNA protein (hTERP). In this collection, Shliapina et al. further our understanding of hTERP role in autophagy regulation. Using hTERP knock-out and over-expression models, the authors showed that hTERP is involved in the regulation of AMPK and mTORC1 activity. Although more work is needed to fully understand the role of hTERP, it is a pinnacle example of how a deeper characterization of the human proteome is essential to truly decipher cellular and molecular pathways.

## Developing the Necessary Tools to Explore the Deep Proteome

Ribosome profiling is the major technological advance that revealed pervasive translation throughout the genome in eukaryotes (Ingolia et al., 2011; Chen et al., 2020). Mass spectrometry quickly followed to demonstrate the existence of protein products from these non-canonical translation sites (Menschaert et al., 2013; Samandi et al., 2017). The development of new technologies and methods is necessary to foster the detection of novel proteins originating from non-coding RNAs.

In this collection, Peeters et al. proposed a proteogenomics workflow combining state-of-the-art mass spectrometer (TimsTOF) and machine learning algorithms to improve the detection of functional peptides in samples. The authors focused on the mouse brain and peptides shorter than 100 amino acids. With an enhanced sensitivity and an optimized search of a large database combining OpenProt (Brunet et al., 2019; Brunet et al., 2021) and the sORFs repository (Olexiouk et al., 2016; Olexiouk et al., 2018), this workflow eases the robust identification of non-canonical peptides.

As the field grows, computational resources have emerged. These include repositories of non-canonical open reading frames (such as OpenProt and sORFs used in studies published in this collection) and browsers of large ribosome profiling data collection, such as GWIPS-viz (Kiniry et al., 2018; Michel et al., 2014) and Trips-Viz (Kiniry et al., 2021; Kiniry et al., 2019). As such, Zaheed et al. present a detailed guide on using GWIPS-Viz and Trips-Viz to explore evidence of translation of allegedly non-coding RNAs. As an example, the authors identify the coding potential of the previously misannotated as lncRNA *LINC00116*. The latter was recently shown to encode the mitoregulin protein and reannotated as the MTLN mRNA (Chen et al., 2018) and thus act as a positive control in the method overview from Zaheed et al.

## Concluding Remarks

The field is still young and this collection highlights recent discoveries, novel technologies and avenues for research. All of these are necessary steps to move away from serendipitous discoveries into systematic explorations of the coding potential of eukaryotic “non-coding” RNAs. This unexplored reservoir of functional proteins might hold the key to a better understanding of cellular and molecular mechanisms.
